# Discovery and Development of One Monomer Molecularly Imprinted Polymers (OMNiMIPs)

**DOI:** 10.3390/polym17172359

**Published:** 2025-08-30

**Authors:** Danielle S. Meador, Stephanie S. Houck, David A. Spivak

**Affiliations:** 1 Department of Chemistry, Louisiana State University, Baton Rouge, LA 70803, USA; daniellemeador@gmail.com; 2 Parkersburg High School, 2101 Dudley Ave, Parkersburg, WV 26101, USA; shouck1@gmail.com

**Keywords:** molecular imprinting, OMNiMIP, crosslinker, MIP, enantioselective, CSP, molecular recognition, artificial receptor

## Abstract

Molecularly imprinted polymers (MIPs) are polymeric receptors for a targeted template molecule that are traditionally formed using a combination of functional monomers and crosslinkers. While investigating novel crosslinkers for MIPs, one of these (2-(methacryloylamino)ethyl-2-methylacrylate (referred to as N,O-bismethacryloyl ethanolamine or “NOBE”)) performed better when used alone versus in combination with other monomers. This introduced the concept of one monomer molecularly imprinted polymers, given the acronym OMNiMIPs, and prompted studies provided in this report that clarify OMNiMIPs have fundamental differences compared to traditionally formulated MIPs. Enantioselectivity studies using BOC-L-tyrosine as a standard template showed that NOBE OMNiMIPs afforded higher-performing MIPs compared with traditional MIPs, have significantly higher binding capacities, and have an internal hydrogen-bonded crosslinking structure that contributes to the morphological stability of the binding site structure. Based on the adventitious discovery of NOBE OMNiMIPs, new analogs based on the NOBE structure were developed and evaluated for further enhancement of molecular recognition performance and novel capabilities of OMNiMIPs. While the majority of the new OMNiMIPs exhibited enantiomeric selectivity toward BOC-L-tyr, improvements were not observed compared with NOBE.

## 1. Introduction

**Scheme 1:**
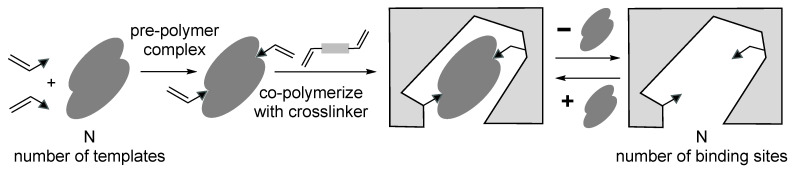
Outline of traditional molecularly imprinting process and theoretical production of binding sites.

Molecularly imprinted polymers (MIPs) are “smart polymers” that are synthesized with the ability to bind specific target molecules [1,2,3,4,5,6], and in some cases elicit catalytic transformations [7,8,9,10]. Using the “lock-and-key fit” paradigm used to describe specific target recognition in enzymes, the molecular imprinting method uses a target template molecule as the “key” around which polymerization will take place to form the “lock”, the matrix which provides binding sites specific to the target molecule (Scheme 1). Early in the development of the molecular imprinting process, researchers used silicate alone as the imprinting matrix [11], which provided both the hydroxyl functional groups for binding interactions with the template, and the shape selective matrix. Later a new approach evolved using organic network polymers, where two or more monomers are pre-complexed to the template to provide specific binding sites in these materials, while the supporting matrix comprised crosslinkers generally regarded as inert toward the template [12]. This strategy has been widely adopted by the scientific community and is generally regarded as the “traditional” method of molecular imprinting. The traditional method of molecular imprinting focuses first on the formation of a pre-polymer complex between a functional monomer (or monomers) and a targeted template molecule (Scheme 1). A second crosslinking monomer that is generally considered inert toward the template is subsequently copolymerized with the pre-polymer complex, providing a rigid matrix that immobilizes the functional monomer(s) in their interactive positions. Thus, the functional monomers are held in a complementary array to the template molecule, and the role of the crosslinked matrix is to provide a non-interactive support for this functional group assembly.

The vast majority of traditionally imprinted macroporous network polymers have been optimized using styrenyl-, acrylate-, and acrylamide-based monomers and crosslinkers formed by radical polymerization methods due to the fact that the neutral radical species does not interfere with the polar interactions between template and functional monomers [6,13]. To a significantly lesser extent, there are a number of non-traditional molecular imprinting strategies that have employed electropolymerization [5,14,15,16], phase inversion [17,18], inorganic or organometallic matrices [19,20,21], and post-polymerization curing [16,22,23,24]. These non-traditional methods have not been studied as thoroughly, and may involve different mechanisms and materials properties compared with traditional MIPs; therefore, the focus of this study will be on molecularly imprinted microporous network polymers.

In contrast to the “traditional” molecular imprinting method, which uses combinations of functional monomer(s) and crosslinker(s), a variant of the molecular imprinting process that uses a single crosslinking monomer has been reported to exhibit superior binding and selectivity when it is used as the only monomer, with the important qualification that performance decreases when utilized in conjunction with other monomers. Imprinted polymers that perform best when only one monomer is utilized are referred to as “OMNiMIPs” which is an acronym for one monomer molecularly imprinted polymers [25,26,27,28]. During evaluation of new crosslinkers for traditional molecular imprinting, the crosslinker N,O-bismethacryloylethanolamine (NOBE) was found to significantly enhance enantioselectivity in MIPs (compared with the use of the crosslinker ethyleneglycol dimethacrylate (EGDMA)) while using methacrylic acid (MAA) as the functional monomer [29]. More exciting, however, was the unexpected finding that a control MIP made with only NOBE, without any other functional monomer, actually provided significantly better enantioselectivity performance compared with MIPs that were formulated in the presence of functional monomer(s) [30].

From these findings, and subsequent successful examples of OMNiMIPs, a question arises as to whether OMNiMIPs are merely a variant of traditional molecular imprinting or whether the OMNiMIP approach is fundamentally different from traditional MIP methods. The research presented here provides evidence that OMNiMIP materials are fundamentally different from traditional MIPs and addresses the underlying factors that lead to low non-specific binding found for the NOBE based OMNiMIPs. In addition, the design, synthesis and evaluation of new crosslinking monomers based on the lead molecular structure of NOBE has been investigated toward novel OMNiMIPs with improved performance.

## 2. Materials and Methods

### 2.1. Materials

Chemicals were purchased from Aldrich, unless otherwise indicated and used without further purification. All solvents were obtained from Fisher Scientific (Pittsburgh, PA, USA) and used without purification. Ethylene glycol dimethacrylate (EGDMA, Polysciences; Warrington, PA, USA) was distilled under vacuum at 94 °C over boiling chips before use. Distillation of methacrylic acid (MAA, Aldrich; Saint Louis, MO) was carried out over molecular sieves (80 °C) prior to polymerization. NOBE (**1**) and NAG (**7**) were synthesized according to published protocols [31].

Reactions were performed in dry glassware under a nitrogen atmosphere. The reactions were checked intermittently by TLC using 0.25 mm Macherey Nagel silica gel glass plates (60F-254), with the adsorbed species visualized by UV light, by iodine, or by staining with molybdophosphoric acid with subsequent heating. Column chromatography was performed using 40–63 μm, 60 Å SiliaFlash irregular silica gel from SiliCycle (Baton Rouge, LA, USA).

#### 2.1.1. Photochemical Polymerization Conditions

For traditional EGDMA/MAA MIPs, 0.27 g (0.96 mmol) of BOC-L-tyrosine was dissolved in 4.0 mL acetonitrile. To this solution, 3.0 g (15.0 mmol) EGDMA, 0.33 g (3.87 mmol) MAA, and 0.051 g (0.30 mmol) AIBN was added. For OMNiMIPs, a solution of BOC-L-tyrosine (0.27 g, 0.96 mmol) and AIBN (0.051 g, 0.30 mmol) in 4.0 mL acetonitrile was added to the appropriate amount of crosslinker to provide OMNiMIPs with 5.0 mol% template loading.

The solutions were split evenly and put into two 8 mm × 94 mm tubes with 2 mm thick glass walls and screw top caps. Nitrogen gas was bubbled into the mixture for 5 min to purge the solution, then the tube was sealed with a cap that was further sealed with Teflon tape followed by parafilm. The tubes were then placed into a rack that was part of a photochemical reactor which was immersed in a constant temperature bath. A standard laboratory UV light source (a Canrad-Hanovia medium pressure 450 W mercury arc lamp) jacketed in a borosilicate double-walled immersion well was placed next to the rack holding the sample tubes. The polymerization was photochemically initiated and allowed to continue for 8 h at 20 °C, while the temperature was maintained by both the cooling jacket surrounding the lamp and the constant temperature bath holding the entire apparatus. The polymer was removed by breaking the tube open and the template was desorbed in methanol by Soxhlet extraction for 2 days. After extraction, the polymers were subjected to grinding and sieving to 25–37 µm in size, then packed into 100 × 2.1 mm stainless steel columns, and their imprinting performance was analyzed by HPLC.

#### 2.1.2. Thermal Polymerization Conditions

All formulations and workup conditions were the same as in the photochemical polymerization formula preparation, with the exception that the sample mixtures were polymerized by insertion into a heated oil bath set at 80 °C for 8 h.

#### 2.1.3. NAG OMNiMIP Polymerization Conditions

After photo-initiated polymerization, the NAG (**7**) formulated OMNiMIP exhibited a gel like consistency, and at the top remained a small amount of isolated solvent. This gel was not solid enough to serve as a stationary phase for HPLC, so further polymerization was required. The viscous material was subsequently treated with another 1 mol% AIBN initiator in 3 mL acetonitrile, purged with nitrogen for 5 min, and placed in a heating bath at 80 °C for 2 days until a dark yellow network solid was formed. The resulting solid was further processed in the same manner delineated in Section 2.1.1 for chromatographic evaluation.

### 2.2. Chromatographic Experiments

The polymers were sized by grinding with a mortar and pestle and passed through U.S.A. Standard Testing Sieves (VWR) to the size range 25–38 μm. The particles were packed as a slurry into stainless steel columns (length, 10.0 cm, i.d. 2.1 mm) to full volume, using a Beckman 1108 Solvent Delivery Module (Beckman-Coulter, Brea, CA, USA), for HPLC experiments, and equilibrated online. HPLC analyses were carried out isocratically at 21 °C (room temperature) using a Hitachi L-7100 pump (Schaumburg, IL, USA) with a Hitachi L-7400 detector (Schaumburg, IL, USA). The flow rate used was 0.1 mL/min, using the mobile phase 99/1 acetonitrile/acetic acid. Sample injections were 5 μL of 1 mM BOC-L-tyrosine or 1 mM BOC-D-tyrosine with peaks detected at 260 nm. The dead volume was established using acetone as an inert substrate. The separation factors (α) of the BOC-tyrosine enantiomers were measured as the ratio of capacity factors (k′_L_/k′_D_). The capacity factors were calculated using the equation: k′ = (R_v_ − D_v_)/D_v_, where R_v_ is the retention volume of the analyte, and D_v_ is the dead volume.

### 2.3. Synthetic Procedures

#### 2.3.1. Synthesis of (Methacryloylamino)Propyl Methacrylate (**4**)

Compound **4** was synthesized by cooling a solution of propanolamine (**2**, 3.98 g, 0.053 mol) in 150 mL DCM to 0 °C, followed by addition of DMAP (0.73 g, 0.006 mol) and MAA (**3**, 4.98 g, 0.058 mol). Upon addition of DCC (11.9 g, 0.058 mol), the mixture was slowly warmed to room temperature and stirred for 28 hr. The DCU was vacuum filtered off, and the organic layer washed with 1 M HCl (aq) (2 × 50 mL) and saturated NaHCO_3_ (aq) (2 × 50 mL). The organic solution was dried using MgSO_4_, and the filtered solution was evaporated to yield an oil, followed by purification using column chromatography employing 50/50 EtOAc/hexanes providing an overall 70% (7.8 g) yield. ^1^H NMR (CDCl_3_, 400 MHz) δ ppm 6.63 (br, 1H), 6.05 (d, 1H, *J* = 3.9 Hz), 5.91 (d, 1H, *J* = 4.1 Hz), 5.55 (d, 1H, *J* = 3.9 Hz), 5.41 (d, 1H, *J* = 4.1 Hz), 4.32 (t, 2H, *J* = 7.1 Hz), 3.21 (t, 2H, *J* = 6.7 Hz), 1.91 (tt, 2H, *J* = 7.1, 6.7 Hz), 1.87 (3H, s), 1.81 (3H, s). ^13^C NMR (CDCl_3_, 100MHz) δ ppm 18.0, 18.3, 28.9, 37.8, 65.3, 119.9, 125.6, 136.1, 139.6, 167.5, 169.0.

#### 2.3.2. Synthesis of Methacrylamidomethyl Methacrylate (**6**)

A solution of N-(hydroxymethyl)-2-methylacrylamide (**5**, 7.78 g, 0.043 mol) in 5 mL water was added to a solution of methacrylic acid (**3**, 4.0 g, 0.047 mol) in 5 mL DMF. To the overall solution, DCC (9.6 g, 0.047 mol) and DMAP (0.57 g, 0.0047 mol) were added, and the reaction stirred for 48 h. The solution was dried under vacuum, and the solid resuspended in 25 mL DCM, after which the remaining solid DCU was filtered off. The remaining solution was washed with 2 × 25 mL of 1M HCl, followed by 2 × 25 mL of saturated NaHCO_3_. After evaporation, column chromatography was employed to purify the crude product using 60/40 EtOAc/hexanes yielding 52% (0.022 mol, 4.09 g) of the product **6**. ^1^H NMR (CDCl_3_, 400 MHz) δ ppm 6.8 (br, 1H), 6.06 (d, 1H, *J* = 3.8 Hz), 5.92 (d, 1H, *J* = 4.1), 5.53 (d, 1H, *J* = 3.8 Hz), 5.42 (d, 1H, *J* = 4.1 Hz,), 5.05 (s, 1H), 1.86 (s, 3H), 1.86 (s, 3H). ^13^C NMR (CDCl_3_, 100 MHz) δ ppm 18.3, 18.8, 69.8, 121.1, 126.6, 137.2, 140.6, 168.3, 171.0.

#### 2.3.3. Synthesis of 2-Acrylamidoethyl Acrylate (**10**)

The 2-acrylamidoethyl acrylate (**10**) synthesis was adapted from a previous literature procedure [32]. Ethanolamine (**8**, 1.0 mL, 0.017 mol) in 100 mL DCM was cooled to 0 °C followed by addition ofacrylic acid (**9**, 2.5 mL, 0.036 mol) and DMAP (0.081 g, 0.0036 mol) in 25 mL DCM. After 10 min, DCC (7.5 g, 0.036 mol) was added dropwise in 25 mL DCM, and the mixture was allowed to cool to room temperature, followed by stirring for 3 days. After the DCU was filtered off, the organic layer was extracted using 1 M HCl (2 × 75 mL) and saturated NaHCO_3_ (3 × 75 mL), and the organic layer was dried over MgSO_4_ and filtered. The organic layer was evaporated, and the crude product was purified by column chromatography using 50/50 to 75/25 EtOAc/hexanes to give a yellow oil providing a 43% (1.20 g) yield of **10**. ^1^H NMR (CDCl_3_, 400 MHz) δ ppm 6.50 (br, 1H), 6.38 (dd, 1H, *J* = 17.3, 1.3 Hz), 6.24 (dd, *J* = 17.1, 1H, 1.5 Hz), 6.09 (m, 2H), 5.82 (dd, *J* = 10.4, 1H, 1.4 Hz), 5.60 (dd, *J* = 10.2, 1H, 1.6 Hz), 4.25 (t, 2H, *J* = 5.4), 3.60 (q, 2H, *J* = 5.5 Hz). ^13^C NMR (CDCl_3_, 100 MHz) δ ppm 38.7, 63.2, 126.6, 127.9, 130.6, 131.4, 165.8, 166.2.

#### 2.3.4. Synthesis of 2-Acrylamidoethyl Methacrylate (**12**)

Hydroxyethyl acrylamide (**11**, 2.12 g, 0.018 mol) was cooled to 0 °C in 75 mL DCM, followed by addition of methacrylic acid (**3**, 1.91 g, 0.022 mol) and DMAP (4.8 mg, 0.039 mmol) combined in 25 mL DCM. DCC (4.46 g, 0.022 mol) dissolved in 25 mL DCM was subsequently added after 5 min, after which the reaction was allowed to cool to room temperature and stirred for 2 days. The DCU was filtered off, and the solution extracted using 0.5 M HCl (4 × 75 mL) and saturated NaHCO_3_ (aq) (4 × 75 mL), and the organic layer dried over MgSO_4_. After filtration and evaporation, column chromatography was used to purify the crude product using 50/50 EtOAc/hexanes to afford 1.78 g (53% yield) of pure product (**12**). ^1^H NMR (CDCl_3_, 400 MHz) δ ppm 6.60 (br, 2H), 6.27 (dd, 1H, *J* = 17.0, 1.5 Hz), 6.11 (m, 3H), 5.65 (t, 1H, *J* = 8 Hz), 5.58 (t, 1H, *J* = 4 Hz), 4.27 (t, 2H, *J* = 5.4 Hz), 3.64 (q, 2H, *J* = 5.5 Hz), 1.93 (s, 3H). ^13^C NMR (CDCl_3_, 100 MHz) δ ppm 18.3, 38.8, 60.4, 63.4, 126.7, 130.6, 135.9, 165.73, 167.5.

#### 2.3.5. Synthesis of 1,3-Dimethacrylamidopropan-2-yl Methacrylate (**14**)

1,3-diaminopropan-2-ol **13**, 2.02 g, 0.0224 mol, was dissolved in CHCl_3_ (50 mL) and cooled to 0 °C. MAA (**3**, 6.7 mL, 0.079 mmol) and DMAP (0.96 g, 7.9 mmol) in 30 mL CHCl_3_ were cooled to 0 °C, then added to the previous solution. DCC (14.9 g, 0.072 mol) was added, and the mixture was allowed to come to room temperature and stir for 3.5 days. The solid DCU was filtered off and the organic layer washed with 1 M HCl (4 × 150 mL), then with saturated NaHCO_3_ (aq) (4 × 150 mL). The organic layer was dried over MgSO_4_, filtered, evaporated, and purified by column chromatography of the crude product using 75/25 EtOAc/hexanes to yield 4.28 g, 65% yield product (**14**). ^1^H NMR (CDCl_3_, 400 MHz) δ ppm 6.88 (br, 2H), 6.10 (s, 1H), 5.77 (s, 2H), 5.88 (S, 1H), 5.36 (s, 2H), 4.96 (m, 1H), 3.62–3.34 (m, 4H), 1.97 (s, 6H), 1.90 (s, 3H). ^13^C NMR (CDCl_3_, 100 MHz) δ ppm 18.2, 18.6, 39.2, 71.4, 120.3, 126.6, 135.9, 139.5, 166.7, 169.1.

## 3. Results and Discussion

### 3.1. Theoretical Model Comparison of OMNiMIPs Versus “Traditional” MIPs

Conceptually, for “traditional” molecularly imprinted polymers, each pre-polymer complex between a template and monomeric functional groups forms a binding site in the final imprinted polymer upon removal of the template; and the theoretical total number of binding sites is equal to the number of complexes (Scheme 1). However, the theoretical total number of binding sites is most often much higher than the “effective” number of binding sites calculated from isotherm analyses or template uptake measurements [33,34,35,36,37]. Improvements in MIP performance that use a non-covalent complex strategy to associate the template and functional monomers have been achieved by promoting complex forming conditions such as lower temperatures or using a less polar solvent which favors hydrogen bonding and electrostatic complex formation [38,39,40,41,42,43,44]. More binding sites can also be achieved by using larger amounts of functional monomer and/or template, which increases the number of complexes via LeChatelier’s principle. Better MIP binding affinity for the template/analyte is expected with an increase in the number of binding sites per gram of MIP, due to the increased amount of template/analyte bound per gram of MIP. This is seen in chromatography where more binding sites per gram of polymer translates to increased separation and resolution of compounds. In addition to creating a greater number of complexes, LeChatelier’s principle can also promote higher numbers of functional monomers that bind to a single template [33]. This creates “higher order” complexes, which have stochastically been shown to increase the binding constant and selectivity of a binding site [45].

However, application of LeChatelier’s principle is limited in traditional MIPs due to the fact that a large amount of crosslinker is required to maintain the binding site geometries of the functional monomers. Seminal studies by Sellergren et al. have explored optimization of the amount of crosslinker in non-covalent MIPs while maintaining a fixed amount of template, leading to a generalization that optimization of MIP performance is achieved at approximately 20% functional monomer, leaving 80% required as crosslinker [43]. Loss of selectivity by imprinted polymers having more than 20 mole % functional monomer is attributed to two underlying mechanisms. First, an excess of functional monomer utilized (e.g., MAA) that is not complexed to the template creates randomly formed non-specific binding sites, which lowers the average overall selectivity of the MIP. Second, there is a minimum amount of crosslinker (e.g., EGDMA) necessary to form a rigid enough polymer network that will maintain the fidelity of the binding site. This limits the amount of non-crosslinking functional monomer (e.g., MAA) that can be employed and optimized for formation of the MIP binding sites, which in turn restricts the amount of template that can be used and ultimately limits the number and quality of binding sites in the traditional MIP.

**Figure 1:**
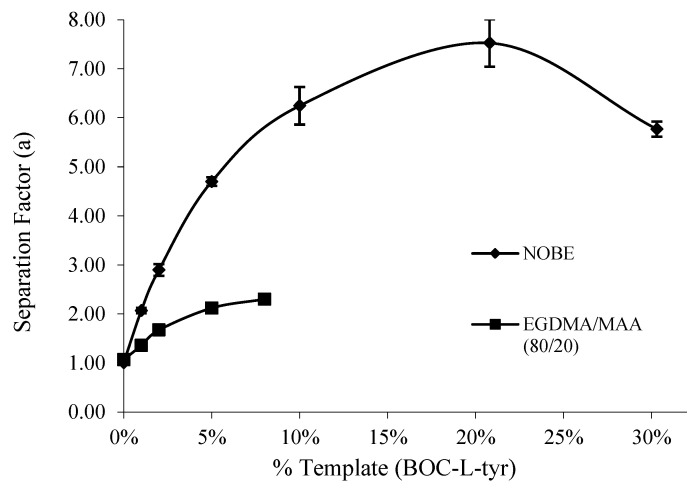
Comparison of enantioselectivity of the NOBE OMNiMIPs versus the traditional EGDMA/MAA MIPs as the template loading increases (template = BOC-L-tyr).

This is further illustrated in a comparison of enantioselective performance of OMNiMIPs versus traditional MIPs as the template loading is increased (Figure 1). Enantioselectivity is the best measure of imprinting performance since the only difference between enantiomers is their three-dimensional array in space, which is exactly what molecular imprinting can differentiate between, while any partitioning differences due to the chemical makeup of any polymer is factored out. To carry out this study, traditionally formed MIPs using 20% MAA as functional monomer and 80% EGDMA crosslinker for enantioselective imprinting of BOC-L-tyrosine (BOC-L-tyr) were synthesized with increasing amounts of template, and were compared to OMNiMIPs exclusively using the NOBE monomer. HPLC was utilized to determine the separation factor (a) of BOC-L-tyr versus tBOC-D-tyrosine, and the results were graphed against the template in Figure 1. The figure shows that not only is enantioselectivity found to be quantitatively better for OMNiMIP at any template loading value, but the separation factor increases considerably, almost exponentially, for the OMNiMIP versus the EGDMA/MAA polymer as template loading is increased. For both MIPs, there is a point where performance no longer increases, and even declines, which is expected to go down because of fewer functional group interactions with the template [43]. Because the BOC-L-tyr is not soluble past 8 mol% loading for the EGDMA/MAA imprinted polymer, the actual decline in performance cannot be charted on Figure 1; however, it is clear that performance is no longer significantly increasing past the 8 mol% loading mark. The OMNiMIP enantioselectivity at 8 mol% template loading is nearly 4-fold higher that of the traditional EGDMA/MAA MIP. Furthermore, the solubility of BOC-L-tyr in the OMNiMIP formulation was 3-fold higher, allowing it to be imprinted with up to 30 mol% template loading.

**Figure 2:**
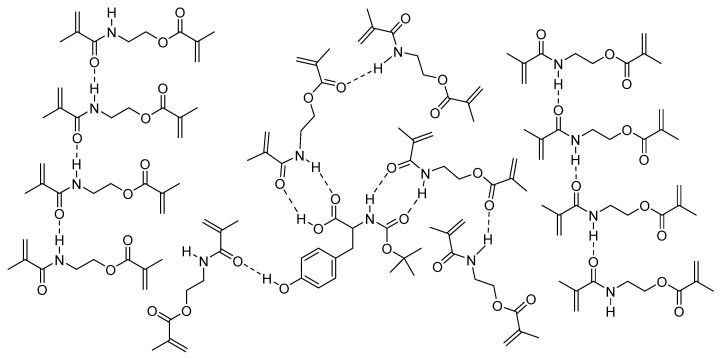
Postulated pre-polymer arrangement of the NOBE crosslinker associated with the BOC-L-tyr template, and self-associated to form a hydrogen-bonded matrix.

To aid in an understanding of the underlying origins of improved performance by the NOBE OMNiMIP, Figure 2 illustrates a possible pre-polymerization complex prior to imprint polymerization. In addition to the BOC-L-tyr template, the only components in the mixture are NOBE and non-interactive solvent, which leaves no alternative but for the NOBE to surround and interact with the BOC-L-tyr template without any competition. It is likely that in this case, the amide groups of the NOBE crosslinker interact with the polar groups of the BOC-L-tyr template via hydrogen bonding and dipole interactions, such as those shown in Figure 2. It is also possible that other NOBE molecules remain in the vicinity of the BOC-L-tyr template via attraction to a polar environment with the amide groups, leading to higher order complexes.

The remaining NOBE crosslinkers that are not in a complex with the template have the opportunity to hydrogen bond with other NOBE monomers that can form a hydrogen-bonding crosslink, which would provide good rigidity that would help maintain the supporting matrix after imprint polymerization. Figure 2 also illustrates the possibility of NOBE crosslinkers binding to NOBE molecules that are part of the pre-polymer complex with the BOC-L-tyr template, creating a second-tier binding site immobilization strategy. Now, as the template loading is increased, the remaining NOBE monomer can form more sites around each of the added template molecules, providing more binding sites per gram of polymer which can increase the binding and enantioselectivity performance. The maximum enantioselectivity for the OMNiMIP appears to occur around 21 mol% template loading as shown in Figure 1, but there is still approximately 3-fold enhanced enantioselectivity at 30 mol% template loading, over the traditional MIP. The high performance of the OMNiMIP at 20 mol%, and even at the 30 mol% template loading is a consequence of a relatively high ratio of the NOBE monomer (acting both as the interactive functional monomer and crosslinker) to the template, which provides five NOBE monomers for each template at 20% loading, and approximately three NOBE monomers for each template molecule. After 30 mol% template loading, the BOC-L-tyr is no longer soluble in the OMNiMIP formulation; however, if solubility were possible, it can be extrapolated that the OMNiMIP could still handle upwards of 50 mol% or more template loading and still outperform the traditional MIP.

**Figure 3:**
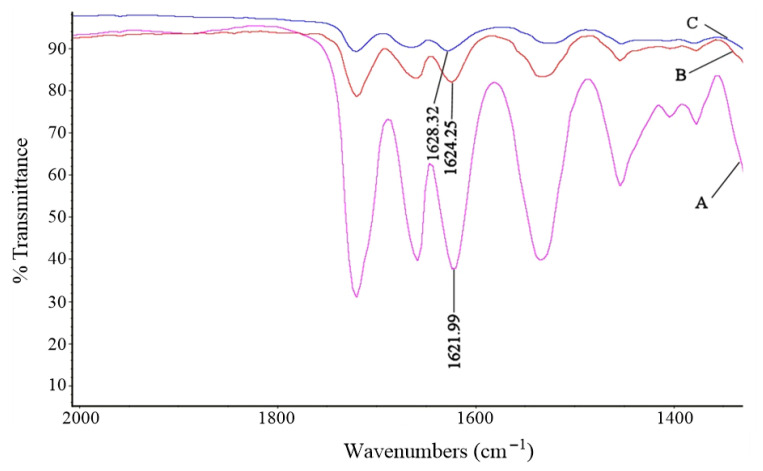
IR cascade spectra as NOBE concentration in Flurolube is increased: (**A**) 4.84 mol/kg, (**B**) 2.28 mol/kg, and (**C**) 1.08 mol/kg.

**Scheme 2:**
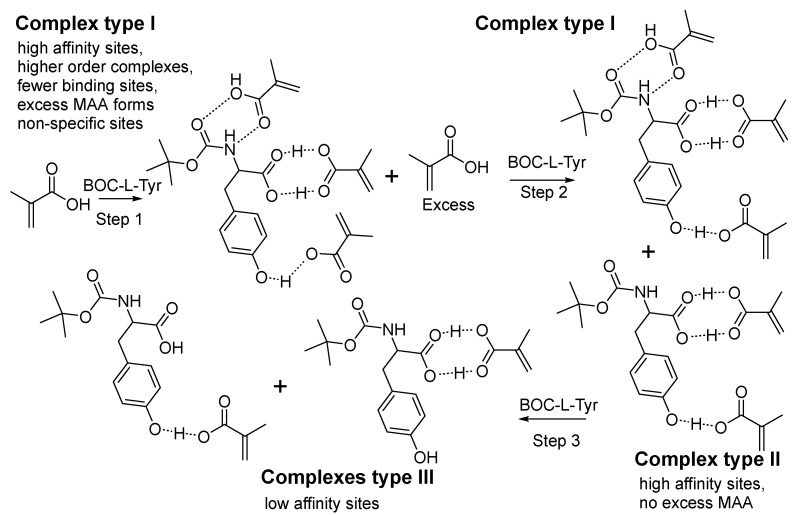
Evolution of pre-polymer complexes as the template (BOC-L-tyr) loading is increased.

On the other hand, the formulation of the traditional MIP provides stoichiometrically different outcomes as template loading is increased, as illustrated in Figure 3. For the traditional EGDMA/MAA imprinted polymer, the enantioselectivity performance increases as template loading increases up to 10 mol%, at which point it appears to level off. The increase in performance is due to the concomitant increase in the number of binding sites in the MIP, as discussed earlier, which provide the maximal number of MAA functional monomers per BOC-L-tyr template molecule during the increase in template loading, estimated to be approximately 1:3 in “Complex type I” in Scheme 2. At 8 mol% template loading, there is approximately a 1:2 ratio of the BOC-L-tyr template to MAA functional monomer, which is represented by “Complex type II” in Scheme 2. Increasing the template loading past 8 mol% is anticipated to created complexes of the sort labeled as “Complex type III” where the ratio of template to MAA functional monomer is on the order of 1:1, a low-order complex that results in poor binding site formation. However, in this example, the BOC-L-tyr is not soluble in the traditional MIP formulation, and the performance values past 8 mol% template cannot be determined. Nonetheless, other reports in the literature following effects of template loading on traditional MIPs have consistently shown binding properties decrease after reaching a maximum, as template loading is increased, citing the same rationale of low functional monomer per template molecule as the template is increased [33,46,47].

**Figure 4:**
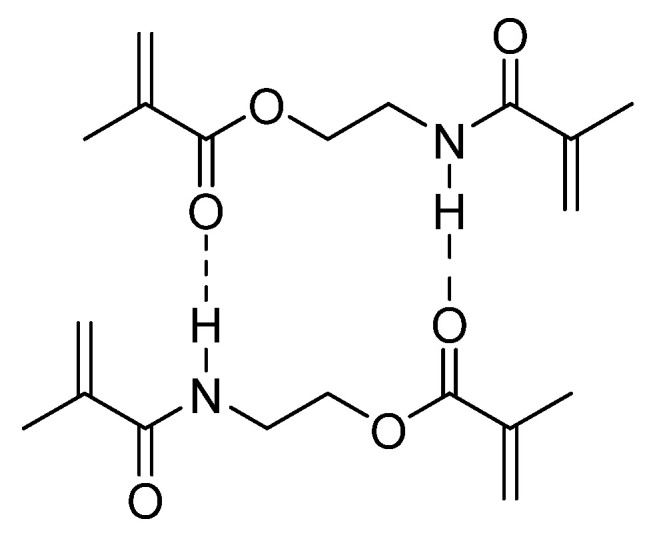
The geometry of NOBE dimer that would show only one IR signal at all NOBE concentrations.

The large increase in performance of the NOBE OMNiMIPs versus traditional MIP materials suggests fundamental differences between these MIPs. The previous discussion pointed out that OMNiMIPs can achieve significantly larger amounts of template loading, leading to more binding sites per gram of polymer that enhance binding outcomes. The reason for this is due to the OMNiMIPs having more template-interactive functional groups that do not suffer limitations according to LeChatelier’s principle. It was also pointed out that the NOBE monomer has the opportunity to hydrogen bond to itself, thereby achieving two goals; first, the excess NOBE that is bound to itself removes free amide functionality that could cause non-specific binding. Second, the self-hydrogen bonding NOBE can create non-covalent crosslinks that further maintain rigidity of the binding site geometry after the template is removed. Figure 2 shows that some of the excess NOBE forms an extended array of hydrogen-bonded crosslinkers; however, it is also possible for the NOBE to dimerize, as shown in Figure 4. The array was posited from a separate study involving IR of NOBE at different concentrations made in a non-interactive solvent Fluorogel (which does not have an IR signal) shown in Figure 4. The figure shows the N-H stretch increasing as the concentration of NOBE is increased, supporting extended array structuring, since the dimer would maintain only one single IR stretch wavenumber at all concentrations of NOBE for dimer formation.

### 3.2. Water Tolerability and Porogen Effects

Further investigation into the role of non-covalent interactions in OMNiMIPs versus traditional EGDMA/MAA polymers was carried out by adding water to the polymer mixtures prior to polymerization. Five MIPs of each type were synthesized, differing only in the amount of water concentration added to the porogen (0,1, 2, 5, and 10% by volume), and the effects on enantioselectivity are shown in Table 1. For both types of MIPs, the addition of 1% *v*/*v* water lowered the enantioselectivity by approximately 20%, which was not surprising and not particularly severe. However, when 2% *v*/*v* water was incorporated into the polymerization formulations, there was a complete loss of all enantioselectivity for the OMNiMIP, whereas the traditional MIP maintained nearly the same enantioselectivity value as the 1% *v*/*v* water added MIP. The precipitous loss of all enantioselective performance by the OMNiMIP synthesized in the presence of 2% *v*/*v* water supports the idea that hydrogen bonding of non-templated monomers that make up the backbone of the polymer matrix contributes extensively to the crosslinking rigidity of the OMNiMIP, in addition to disrupting the template–monomer complexes. A similar observation with NOBE OMNiMIPs has been reported previously where the incorporation of other functional monomers that can hydrogen bond also lowered the enantioselectivity of the OMNiMIPs, which was attributed in part to disruption of the hydrogen bond crosslinks in the OMNiMIP [15]. The only exception noted in the publication showed that methacrylamide as functional monomer did not disrupt the OMNiMIP, indicating that amide group in the monomer participates in the extended array of the hydrogen-bonded matrix around the template binding sites. As the water portions were increased to 5% and 10% *v*/*v* in the MIP formulations, the OMNiMIP, as expected, showed no enantioselecticity compared with the traditional MIP, which still exhibited a small enantioselective bias. This indicates that the water is much more tolerable in the traditional MIPs compared with OMNiMIPs, possible due to stronger electrostatic interactions of template with MAA and the lack of any hydrogen bonding to stabilize the support matrix.

The OMNiMIPs were also formulated with different porogens (using 4 mol% BOC-L-tyr template), which are the solvents in the polymerization that are responsible for controlling the porous properties in the imprinted materials, as shown in Table 2, along with the corresponding enantioselectivity values. The results are in general agreement with majority of the literature reports investigating porogen properties in MIPs, showing aprotic polymer solvents provide higher performance in MIPs, whereas protic solvents or very polar solvents such as DMF provide poor performance in MIPs due to disruption of non-covalent interactions [48].

### 3.3. Temperature Effects

The purpose of this study was to compare the differences in performance of photo-polymerization and thermal-polymerization methods. Thermal polymers were polymerized using a temperature-controlled oil bath set at 80 °C for 8 h, while photopolymerization was performed at 22 °C under a UV lamp for 10 h, and the formulations were the same for both polymers. Table 3 shows that for both types of MIPs, the photopolymerized materials exhibit significantly higher enantioselectivity values for the photopolymerized MIPs versus the thermally polymerized materials, which are consistent with other reports in the literature. This is attributed to the entropic disruption of non-covalent complexes at elevated temperatures versus the entropic promotion of non-covalent complexes that ultimately lead to specific binding sites afforded at the lower temperatures that photopolymerization accommodates. Table 3 also shows the capacity factors for each enantiomer of tBOC-Tyr, with the increase in the capacity factor for the D enantiomer indicating a significant increase in non-selective binding, which is not equally represented by the traditional EGDMA/MAA MIP. This may again be attributed to the decrease in hydrogen bonding in the matrix backbone of the OMNiMIP, leaving free amide functionality that is not templated to cause non-specific binding, also making binding sites less rigid, lowering their selectivity. This is another significant difference between OMNiMIPs and traditional MIPs, also seen in both the water tolerability study and the temperature study, supporting the important role of matrix hydrogen bonding in the performance of NOBE OMNiMIPs.

### 3.4. Design, Synthesis, and Evaluation of New OMNiMIP Materials

#### 3.4.1. Synthesis of OMNiMIP Crosslinkers

**Scheme 3:**
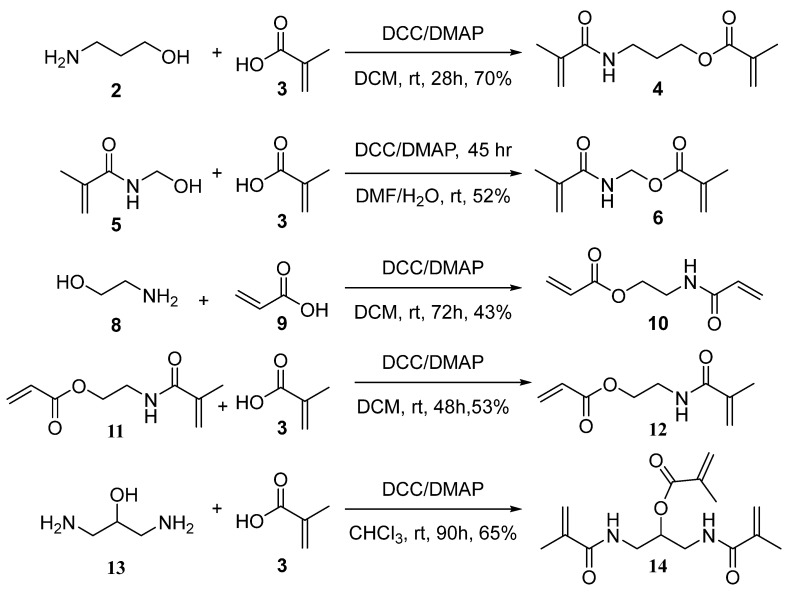
Outline of synthetic routes for new crosslinkers to be evaluated in OMNiMIP formation.

With the surprising discovery of the NOBE crosslinker providing better performance as an OMNiMIP rather than a mixture of monomers, new analogs based on the NOBE structure were developed and evaluated for enhanced molecular recognition performance and novel capabilities. As shown in Scheme 3, a parallel approach was used for the synthesis of the new crosslinkers, which was based on the synthesis of NOBE. In each case, the precursor alcohol or alcohol amine was reacted with methacrylic acid or acrylic acid using dicyclocarbodiimide (DCC) catalyzed with dimethylaminopyridine (DMAP). Yields in most cases were not optimized but provided ample material for testing.

#### 3.4.2. OMNiMIP Performance of Crosslinkers with Different Lengths

Initial experiments toward this goal involved the synthesis of two new crosslinkers in which the length of the center hydrocarbon spacer of the NOBE crosslinker structure is increased or decreased by one methylene unit. Looking at Table 4, entry 2 shows that increasing the length of the center hydrocarbon spacer to the 3-carbon propyl group decreases selectivity to a large degree. The reason for the loss of selectivity introduced by the longer crosslinker has precedent in molecular imprinting, due to the increased flexibility of the monomer, which decreases the rigidity and recognition of the MIP binding sites [44]. It was also found that shortening the NOBE hydrocarbon spacer to a 1-carbon spacer (entry 3 in Table 4) decreased OMNiMIP enantiselectivity relative to NOBE. There could be more than one possible reason for this; for example, hydrogen bonding in the OMNiMIP support matrix may not align properly, or the shorter distance between crosslinks may not provide optimum binding site space. However, when the spacer between polymerizable double bonds was further reduced in length by removing the ester oxygen (entry 4 in Table 4, referred to as N,O-bismethacryoyl glycine, or NAG), enantioslective performance was improved versus the compound in entry 3 of Table 4, but not as good as NOBE. Earlier studies that employed NAG in a traditional MIP in conjunction with MAA as the functional monomer showed better performance than NOBE under the same conditions. Further studies in that report employing thermal polymerization revealed that the vinyl ketone moiety of NAG polymerized before the methacrylamide group, which caused morphological changes and may account for the results presented here. It should be noted that difficulties during photopolymerization were encountered, where instead of formation of a solid polymer monolith, a viscous solution remained. This material was subsequently treated with another 1 mol% AIBN initiator in 3 mL acetonitrile and placed in a heating bath at 80 °C for 2 days until a dark yellow network solid formed. In spite of the difficulties encountered, analysis of this material afforded a good separation factor of 2.4, indicating that this crosslinker has tremendous potential as an OMNiMIP using thermal polymerization. This is a highly significant finding since thermal polymerizations are preferable in an industrial setting. Overall, these studies currently show that the photopolymerized NOBE OMNiMIP provided the best enantioselective performance of an MIP under the conditions tested.

#### 3.4.3. OMNiMIP Performance from Crosslinkers with Modification of NOBE Polymerizable Groups

Having assessed the effects on enantioselectivity on the size of the central group of NOBE, the next study was focused on the nature of the polymerizable double bond groups on either side of NOBE. Two analogs of NOBE were tested in this respect; first, compound **5**, where the methacrylamide groups were replaced with acrylamide groups, and compound **6**, which incorporated an methacrylate group on one side and an acrylamide group on the other (shown in Table 5). The analogs 5 and 6 maintain the necessary amide functionality for interaction with the template, differing only by the polymerizable groups to determine changes in molecular recognition. As shown in Table 4, the performance of both analogs provided OMNiMIPs that could separate enantiomers but were still significantly less than the capability of NOBE. The inferior performance of crosslinkers 5 and 6 is due to the substitution of acrylate moieties in place of the methacrylate moieties of NOBE, which is known to form less rigid polymer matrices as a result of allowing more torsion around the crosslinks. This is supported by the higher enantioselectivity of crosslinker 6 with only one acrylate group, versus crosslinker 5, which incorporates the acrylate moiety on both sides. The greater torsion around crosslinks contributes to increased overall flexibility of the OMNiMIPs formed using crosslinkers 5 and 6, leading to loss of fidelity in the binding site geometries of the OMNiMIPs.

The addition of a third double bond polymerizable group was also investigated, focusing on a novel crosslinker 7 shown in Table 5 entry 4, which incorporates an additional methacrylamide group to the NOBE structure. The additional amide moiety was anticipated to provide additional hydrogen bonding, and more crosslinking could provide a more rigid matrix to improve enantioselective performance. However, compound **7** in Table 4 shows that while an OMNiMIP using the trifunctional crosslinker did provide enantioselectivity, it was still considerably less than that found for the NOBE OMNiMIP. One possible reason could be the OMNiMIP using trifunctional crosslinker 7 that could block interactions with the BOC-L-tyr template. Moreover, the sterics of the third methacrylamide group may prevent the hydrogen-bond induced stacking of the matrix crosslinker, which gives the matrix greater rigidity and maintains backbone structure of the NOBE OMNiMIPs. In addition, the additional methacrylamide group may have provided more non-specific binding interactions to occur that would contribute to a loss of selectivity. Overall, these studies clearly show that the polymerizable groups of crosslinkers play a vital part in OMNiMIP formation.

## 4. Conclusions

The studies reported herein have illuminated fundamental differences in the recently developed OMNiMIP materials compared with the traditional imprinted polymers. First and foremost, it has been shown that optimum-performance OMNiMIP materials, such as those fabricated using NOBE, are found when only a single crosslinking monomer is used. In fact, it was shown that addition of other monomers decreased the performance of OMNiMIPs made using NOBE alone, with the exception of acrylamide, which closely mimics the molecular components of NOBE. This was the initial indicator that OMNiMIPs are intrinsically different from traditional MIPs that rely on a combination of monomers to optimize MIP performance. However, further studies have also pointed to several other important differences between these MIP methods. For example, the blending of template-interactive functionality into a crosslinking format removes limitations in traditional molecular imprinting, which only allows low template loading before binding performance fails. Thus, the OMNiMIP materials are inherently capable of significantly greater template loadings that lead both to highly increased selectivity performance, and a large increase the binding capacity, or total amount of template/analyte that can be bound by the MIP. A third important difference is the role of internal hydrogen bonding of the OMNiMIP crosslinker to itself, when it is not interacting with the template. This achieves the vital goal of eliminating non-templated functional group interactions with analytes, while providing further crosslinking to maintain rigidity of the matrix supporting the actual binding sites.

This was further supported by investigating analogs of NOBE that were not able to achieve the same inter-crosslinker hydrogen bonding evidenced by NOBE. While all analogs developed here did not improve the performance found for NOBE OMNiMIPs that are photochemically polymerized, the NAG analog was found to perform better than NOBE for thermally polymerized OMNiMIPs, which was likely due to differences in the polymerization mechanism. Not only have the NOBE OMNiMIPs provided enhanced binding and selectivity performance over traditional MIPs, but these materials are also synthetically easier in that only one monomer is needed, eliminating complicated optimization of multi-monomer MIPs and problems with incompatible monomers. It should be noted that the NOBE OMNiMIP materials work best for templates that interact by hydrogen bonding, while traditional MIPs can utilize ionic interactions as well as hydrogen bonding, which in some cases provide better performance for the traditional MIPs. Nonetheless, it has been shown that when NOBE is used in place of EGDMA as crosslinker for traditional MIPs, the traditional NOBE imprinted polymer still provides better results than EGDMA.

## Figures and Tables

**Table 1:** Comparison of the effect of increasing water content in the porogen on enantioselectivity between NOBE OMNiMIPs compared with traditional MIPs made with EGDMA/MAA: 80/20.

% H_2_O	Enantioselectivity Separation Factor (α) for NOBE OMNiMIPs	Enantioselectivity Separation Factor (α) for Traditional MIPs
0	4.7	2.2
1	3.9	1.7
2	1.0	1.6
5	1.0	1.3
10	1.0	1.1

**Table 2:** Effects of solvent as porogen on enantioselectivity of NOBE OMNiMIPs.

Entry	Solvent	Enantioselectivity Separation Factor (α) for NOBE OMNiMIPs
1	Acetonitrile	4.7
2	Chloroform	3.70
3	Toluene	2.10
4	Methanol	1.10
5	DMF	1.09

**Table 3:** Chromatographic data comparing photochemical versus thermally initiated polymers.

Entry	Polymer	Type of Polymerization	k′_D_	k′_L_	Enantioselectivity Separation Factor (α)
1	NOBE OMNiMIP	Photochemical	2.13	10.02	4.70
2	NOBE OMNiMIP	Thermal	3.29	6.63	2.02
3	EGDMA/MAA (80/20)	Photochemical	0.23	0.49	2.13
4	EGDMA/MAA (80/20)	Thermal	0.19	0.26	1.37

**Table 4:** Effects of crosslinker length on enantioselectivity.

Entry	OMNiMIP Crosslinker	Enantioselectivity Separation Factor (α)
1	** 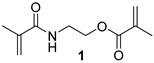 **	4.7
2	** 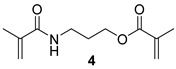 **	1.3
3	** 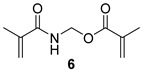 **	1.6
4	** 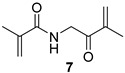 **	2.4

**Table 5:** Evaluation of enantioselectivity for OMNiMIPs synthesized from crosslinkers with modification of NOBE polymerizable groups.

Entry	OMNiMIP Crosslinker	Enantioselectivity Separation Factor (α)
1	** 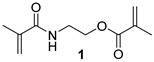 **	4.7
2	** 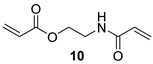 **	1.3
3	** 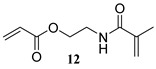 **	1.6
4	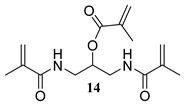	2.4

## Data Availability

Data are available from the corresponding author upon request.

## Abbreviations

The following abbreviations are used in this manuscript:

DCM	Dichloromethane
DCC	Dicyclohexylcarbodiimide
DMAP	Dimethylamino pyridine
EGDMA	Ethyleneglycol dimethacrylate
MAA	Methacrylic Acid
BOC	tert-Butyloxycarbonyl
